# 3D accuracy and clinical outcomes of corrective osteotomies with patient-specific instruments in complex upper extremity deformities: an approach for investigation and correlation

**DOI:** 10.1186/s40001-022-00830-9

**Published:** 2022-10-08

**Authors:** Heide Delbrück, David Christian Weber, Jörg Eschweiler, Frank Hildebrand

**Affiliations:** Department of Orthopedic Surgery, Trauma and Reconstructive Surgery, RWTH Aachen University Medical Center, Pauwelsstr. 30, 52074 Aachen, Germany

**Keywords:** Upper extremity, Osteotomy, Patient-specific implants, Surgical guides, 3D accuracy

## Abstract

**Background:**

Corrective osteotomies of the upper extremities with patient-specific instruments (PSIs) are increasingly used. In this context, the concordance between planning and postoperative 3D radiographs as well as the association between 3D accuracy and clinical outcome has rarely been evaluated. In this pilot study, we aimed to investigate our clinical mid-term outcome and 3D accuracy as well as their possible correlation, including identifying aspects critical to reaching optimal correction results.

**Methods:**

From October 2018 to January 2020, we used PSIs for 12 corrective osteotomies of the upper extremity in 11 bones of 8 patients (congenital or posttraumatic deformities in 2 elbows, 3 forearms, 3 distal radii). In follow-up examination (10–25 months postoperatively), patient satisfaction, grip strength, ROM, VAS, and DASH were evaluated. Three-dimensional radiological accuracy was determined with 3D-reconstructed postoperative CT scans. With the software tool “Part Comparison” of Mimics^®^ Innovation Suite Software/Materialise, surface differences of pre-planned and postoperative 3D models were compared.

**Results:**

Compared to the preoperative situation pain and function were better at follow-up: The average VAS score significantly decreased from 6.5 ± 4.1 cm preoperatively to 2.3 ± 2.6 cm at the follow-up time point (*p* = 0.008). The average DASH score significantly improved, from 48.4 ± 30.9 to 27.0 ± 25.2 (*p* = 0.015). In the part comparison analysis “planned vs postoperative comparison”, significantly more points in percent (= 3D accuracy) were in a −3 mm to 3 mm interval than in the “preoperative vs planned comparison” (87.3 ± 13.8% vs 48.9 ± 16.6%, *p* = 0.004). After surgery, the maximum deviation value over all cases was 4.5 ± 1.1 mm, and the minimum deviation value was − 4.5 ± 1.2 mm vs preoperatively 12.9 ± 6.2 mm (*p* = 0.004) and − 7.2 ± 2.1 mm (*p* = 0.02), respectively. Clinically, in all cases with higher accuracy (> 90%), an improvement of either DASH or VAS or both of > 60% to the preoperative values occurred. There was a significant correlation between accuracy (%) and ΔVAS (*p* = 0.004). There were no method-related complications.

**Conclusions:**

Our data after PSI-based corrective osteotomy in complex deformities of the upper extremity in a limited number of cases indicate a positive correlation between 3D accuracy and clinical outcomes. Examination of 3D accuracy to analyse sources of error in the hole procedure from initial CT scan to end of surgery even in patients with not fully satisfactory clinical results is required for further development of the method to achieve optimal correction results with nearly 100% congruence between the planned and postoperative 3D bone position.

*Trial registration* This retrospective study was registered in the Center for Translational & Clinical Research Aachen (CTC-A) with the number 20-514 on November 20, 2021

## Background

Correction of complex three-dimensional (3D) deformities of the upper extremity with plain radiographs and CT scans, including intraoperative evaluation represents a challenging approach. Additionally, intraoperative evaluation of the correction result is well known to be difficult [1–6].

Computer-aided 3D surgery planning with the implementation of 3D printed patient-specific templates, including the information for drill, cutting, and re-positioning in the different bony parts (the so-called patient-specific instruments = PSIs), seems to be a promising tool to optimize the intervention results in these cases. So far, a limited number of studies, case series, and case reports considering upper extremity deformities have investigated the relevance of this technique and found promising results [1–4, 6–13]. However, the comparability of these studies is limited due to the various methods, software, and hardware tools used for 3D planning and printing. Furthermore, for postoperative evaluation of the accuracy of correction results, no consistent approach regarding the use of 2D and 3D data exists. Many studies have measured postoperative outcomes using 2D X-ray images [3–5, 9]. Since preoperative planning was based on 3D data to achieve greater correction accuracy, postoperative control should also be performed with 3D data. In this context, some studies have used postoperative CT data [1, 4, 6, 7, 10, 12, 13], but, in sum, the outcome evaluation is inconsistent and follows no standards. Some analyse the accuracy by measuring angle and distance differences in standard coordinate systems [1, 4, 10], whereas others calculate the Euler angle to investigate the rotational corrections [6, 7, 12, 13]. Furthermore, only very rarely is the association between 3D accuracy and clinical outcome associated [5].

This retrospective observational pilot study aimed to evaluate 3D accuracy by comparing the planned vs postoperative bone position with an iterative closest point analysis to associate the determined accuracy with the clinical outcome. As far as we know, we are the first to evaluate data after corrective osteotomies in the upper extremity with the software tool “Part Comparison” of Mimics^®^ Innovation Suite Software/Materialise. Furthermore, for the planning and printing of PSIs, we used the often applied [1, 4, 12] service of the company Materialise (Leuven, Belgium), and so we especially examined the accuracy of this commercial workflow.

## Methods

### Patients

From October 2018 to January 2020, all 8 patients on whom we performed a corrective osteotomy with PSI in the upper extremity (5 male and 3 female) were included in this retrospective study. Data were collected from the medical records and the imaging available. The age of patients was between 15 and 64 (32.8 ± 18.9) years. A total of 12 osteotomies in 11 bones were carried out. Individually planned and 3D-printed PSIs for upper extremity surgery were used. Two surgeries were performed around the elbow due to posttraumatic deformities, three were cases of forearm deformities (two posttraumatic, one due to cartilaginous exostosis), and three were cases of distal radius deformities (two posttraumatic, one Madelung deformity). Further details of the included patients are provided in Table 1 and the time point of CT scan in the section “Radiological postoperative evaluation”. The patients were regularly examined during our outpatient consultation hours as part of postoperative follow-up care until the complete bone consolidation of the osteotomies had occurred, and the original daily activity could be resumed (follow-up 14.4 ± 5.0 months).

**Table 1:** Patient details

Patient	Gender	Age (years)	Reason for deformity	Follow-up (months)	Problem	Aim of surgery	Location of osteotomy
Elbow							
1	Male	37	An untreated childhood elbow injury	16	Cubitus varus 30°, radial head subluxation, ulna recurvation, and shortening	Pain relief in the radial elbow, reconstruction of normal anatomy (cosmetic reasons)	Distal supracondylar humerus, proximal ulna
2	Male	18	An untreated supracondylar humeral fracture as an 8-year-old	18	Cubitus varus 20°	Pain relief radial elbow, reconstruction of normal anatomy (cosmetic reasons)	Distal supracondylar humerus
Forearm							
3	Male	18	An untreated forearm fracture as a 9-year-old	24	Pronation contracture of the forearm	Reconstruction of neutral forearm rotation, restore forearm rotation	Diaphyseal radius and ulna
4	Male	15	Ulnar shortening and radius bowing with radial head dislocation due to hereditary multiple exostoses	14	Ulna recurvation, prominent radial head luxation, extension deficit of elbow 30°	Improvement of forearm rotation and elbow extension, radial head reposition	Proximal ulna
5	Male	47	Malunion of forearm fracture 13 years prior	9	Malunion diaphyseal radius with ulna plus variant and prominent dorsal dislocation of the distal ulna	Pain relief distal radioulnar joint, reconstruction of normal anatomy, improvement in forearm rotation	Radius double osteotomy, distal diaphyseal ulna
Distal radius							
6	Female	48	Malunion of distal radius fracture 1 year prior (surgical) treatment)	14	Malunion distal radius with shortening and flexion	Pain relief, improvement of dorsal extension, and supination	Distal radius
7	Female	64	Malunion of distal radius fracture 4 months prior (conservative treatment)	10	Malunion distal radius with 20° dorsal tilt, ulna plus variant, decrease of ulnar inclination	Pain relief, reconstruction of normal anatomy	Distal radius
8	Female	15	Madelung deformity	10	An increased ulnar inclination angle	Pain relief distal radioulnar joint, reconstruction of normal anatomy	Distal radius

### Planning of surgery and guides

**Fig. 1:**
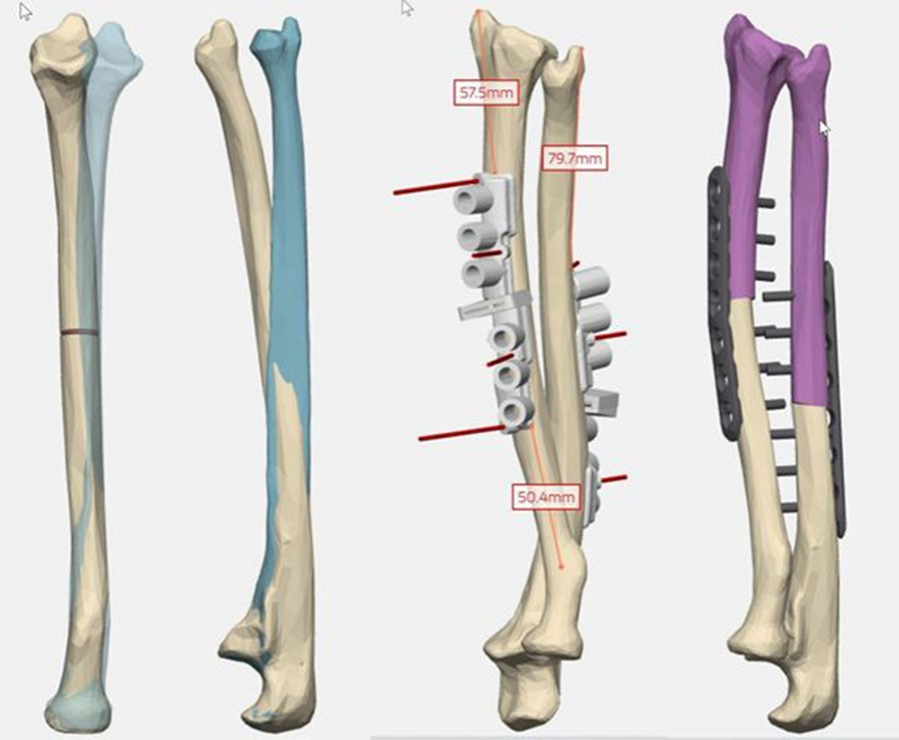
3D-supported planning of the corrective osteotomy of case 3. Left: diseased (ivory) and mirrored healthy opposite side (blue) aligned proximally. The planned osteotomy height is already drawn in on the radius. Right: position of the planned osteotomy, drilling and cutting guides, and outcome model

Depending on the location of the deformity, CT scans of the whole affected bone and the opposite side were obtained (upper and lower arm at both sides in case 1, upper arm on both sides in case 2, forearm on both sides in cases 3–8). The examination was carried out according to the specified scan protocol from Materialise, Leuven, Belgium (slice thickness humerus 1.25 mm, forearm 0.625 mm) [14]. Segmentation and 3D reconstruction were carried out by the company’s planning service. For an ideal reconstruction of the pathological side and as a reference, the healthy opposite side was then mirrored (Fig. 1); in the case of bilateral disease (case 8), an age-appropriate reference model was used. (We included this patient despite this methodological difference because there is no other way than to correct bilateral deformities to the norm values of the reference population. For determination of 3D accuracy it should not be relevant at most for clinical statements.) The surgeon determined the optimum osteotomy height, type of plate, and position so that the side to be operated on corresponded to the opposite side of the reference model. Planning and 3D printing of the PSIs were then carried out by Materialise. A comprehensive planning protocol was drawn up for each step of surgery [15].

### Surgical procedure

**Fig. 2:**
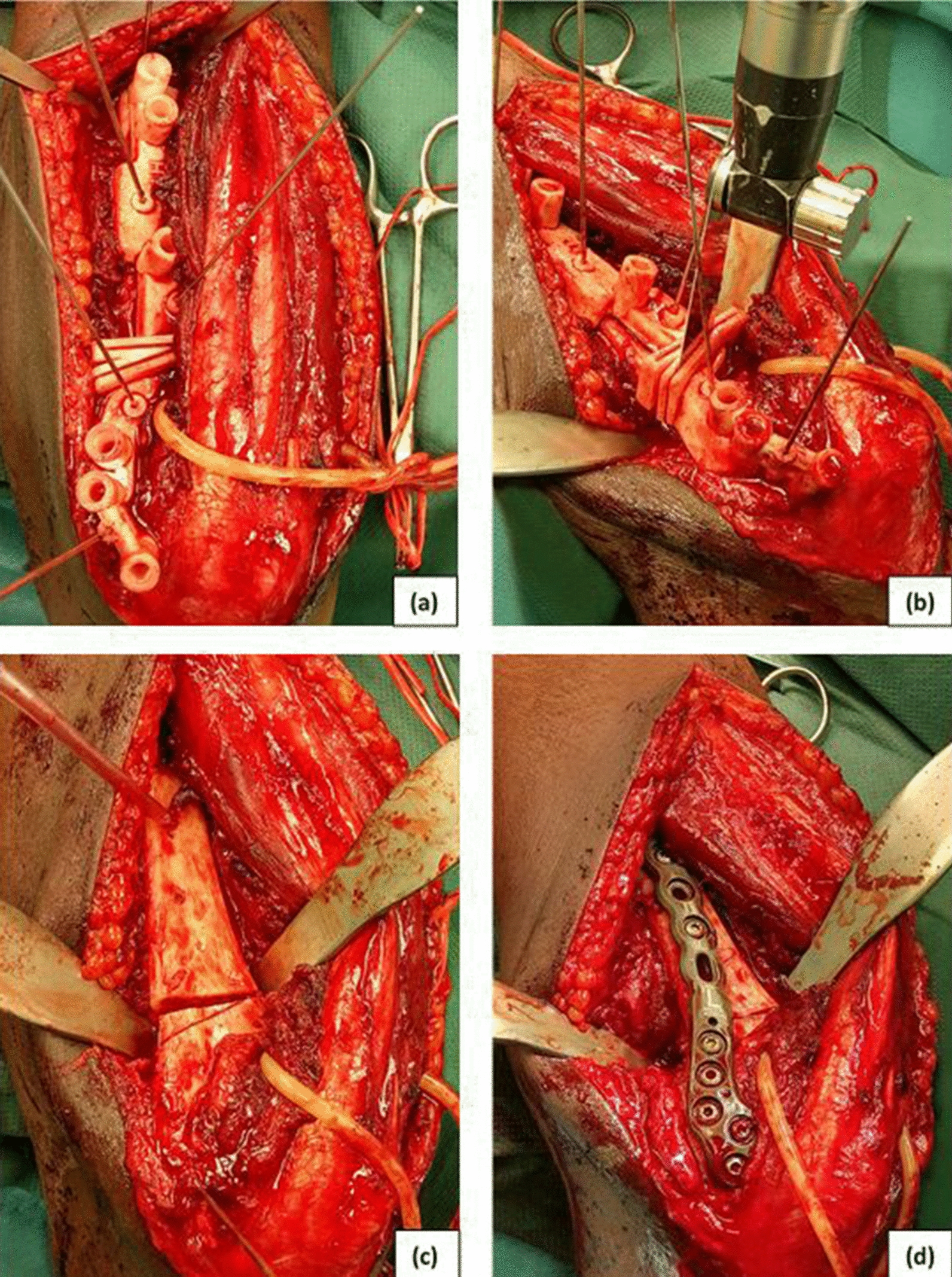
Surgical procedure (case 2, view to distal dorsal humerus): **a** correct placement of the drill and cutting guide on the bone surface; **b** after drilling screw holes, the osteotomy is performed; **c** after removing the guide; **d** completed correction and plate fixation

After a sufficient surgical exposition of the osteotomy region, the PSIs were placed on the bone surface in the order of preoperative planning (Fig. 2).

After drilling the screw holes through the PSIs, the osteotomy was performed with help of the PSIs themselves, and then the selected plate was placed. By placing the screws through the predrilled holes, a reduction with the desired correction was achieved. The screw lengths were also planned. In patients with multiple exostoses, ulnar shortening and radius bowing with radial head dislocation is a common situation. Gradual ulnar lengthening is a safe and reliable procedure for this situation [16]. We analysed the three-dimensional extension of radius bowing in case 4. With a maximum of 10°, it was not so pronounced to consider a radius osteotomy. The placement of the bone screws of the external unilateral lengthening fixator and the ulnar osteotomy was done with the help of the PSI so that in addition to the ulnar lengthening, its axis could also be corrected. The same surgeon with surgical experience of more than 15 years was responsible for planning and performing the surgeries in all 12 osteotomies.

### Clinical and patient-reported postoperative evaluation

At the time of follow-up (see Table 1: patient details), the range of motion (ROM) and grip strength of both sides were measured by an experienced resident physician. For ROM measurement, a goniometer and, for grip strength, a dynamometer (Hydraulic Hand Dynamometer, SH5001, SAEHAN Corporation, Korea) were used [17]. According to the user manual for the dynamometer, the values measured were adjusted for sex, age, and handiness-dependent clinical norms according to Mathiowetz et al. [18]. Pain (Visual Analogue Scale = VAS; 10 cm ruler was presented: 0 cm no pain, 10 cm maximum imaginable pain) and Disabilities of Arm, Shoulder and Hand (DASH) score for current and preoperative time points were inquired [19, 20]. Furthermore, patient satisfaction was evaluated (possible answer options: very satisfied, satisfied, neither satisfied nor dissatisfied, dissatisfied, very dissatisfied).

### Radiological postoperative evaluation

In addition to routinely conducting control X-rays in the postoperative course until bony consolidation, we carried out CT examinations according to the routine postoperative treatment plan of our clinic. In cases 2 and 4, due to the excellent clinical results (both patients had a VAS and DASH of 0 after surgery and were very satisfied with it), CT was not done due to radiation protection reasons. Therefore, only postoperative CT data of 6 patients were available for the verification of the 3D accuracy within this retrospective study.

### Angle measurements and part comparison with pre- and postoperative CT

**Fig. 3:**
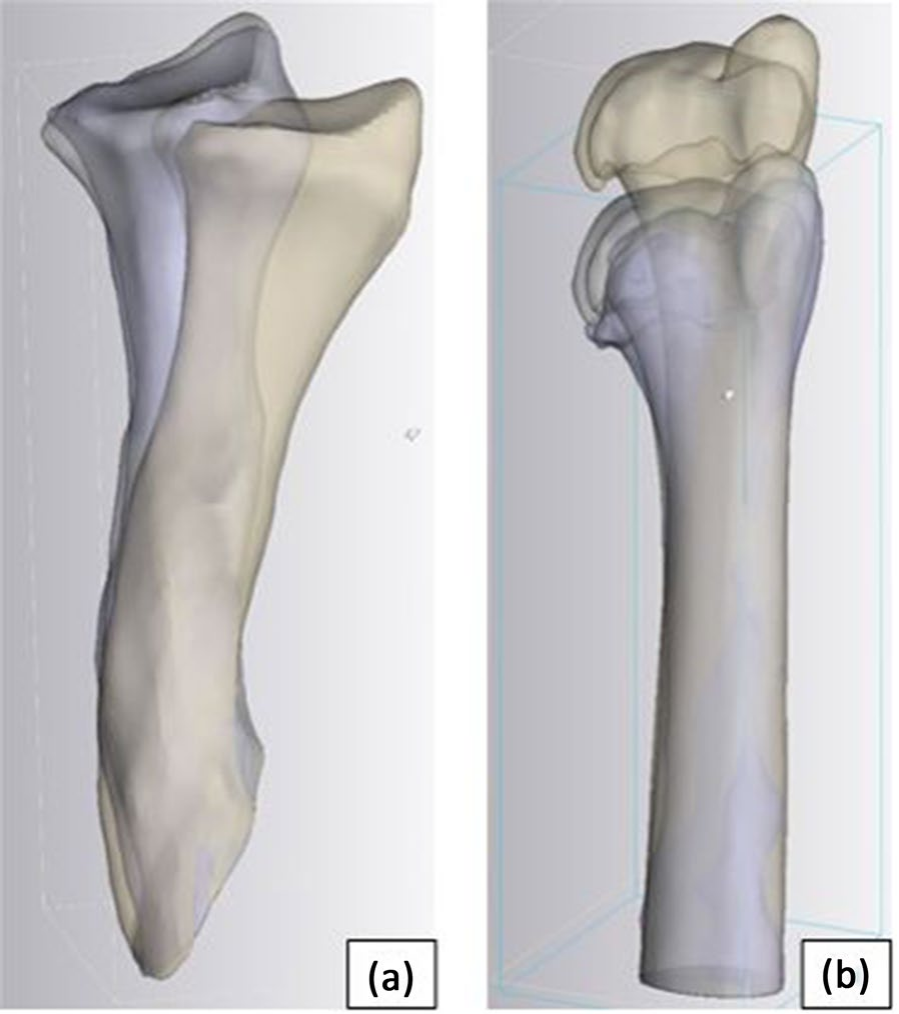
Parts distal to osteotomy of patient 5. Colours: ivory = before surgery, grey = planned outcome, violet = real outcome. **a** radius distal to the proximal osteotomy; **b** ulna distal to the proximal osteotomy

The software Mimics Medical (V.24.0) and Materialise 3-Matic Medical (V.16.0) (both Materialise, Leuven, Belgium) were used for the analysis performed by the surgeon. Pre- and postoperative CT scans were semi-automatically segmented for each patient by an expert. This was done in 6 cases (9 bones) where both were available. With the software tools N-Points Registration and Global Registration, the preoperative, planned, and postoperative parts proximal of the osteotomy (respectively, distally of the ulnar osteotomy in case 1) were aligned exactly to each other. Then, we carried out the part comparison analysis for the part distally (in the ulna in case 1 proximally) of the osteotomy by comparing both the postoperative and the preoperative position with the planned position (Fig. 3).

**Fig. 4:**
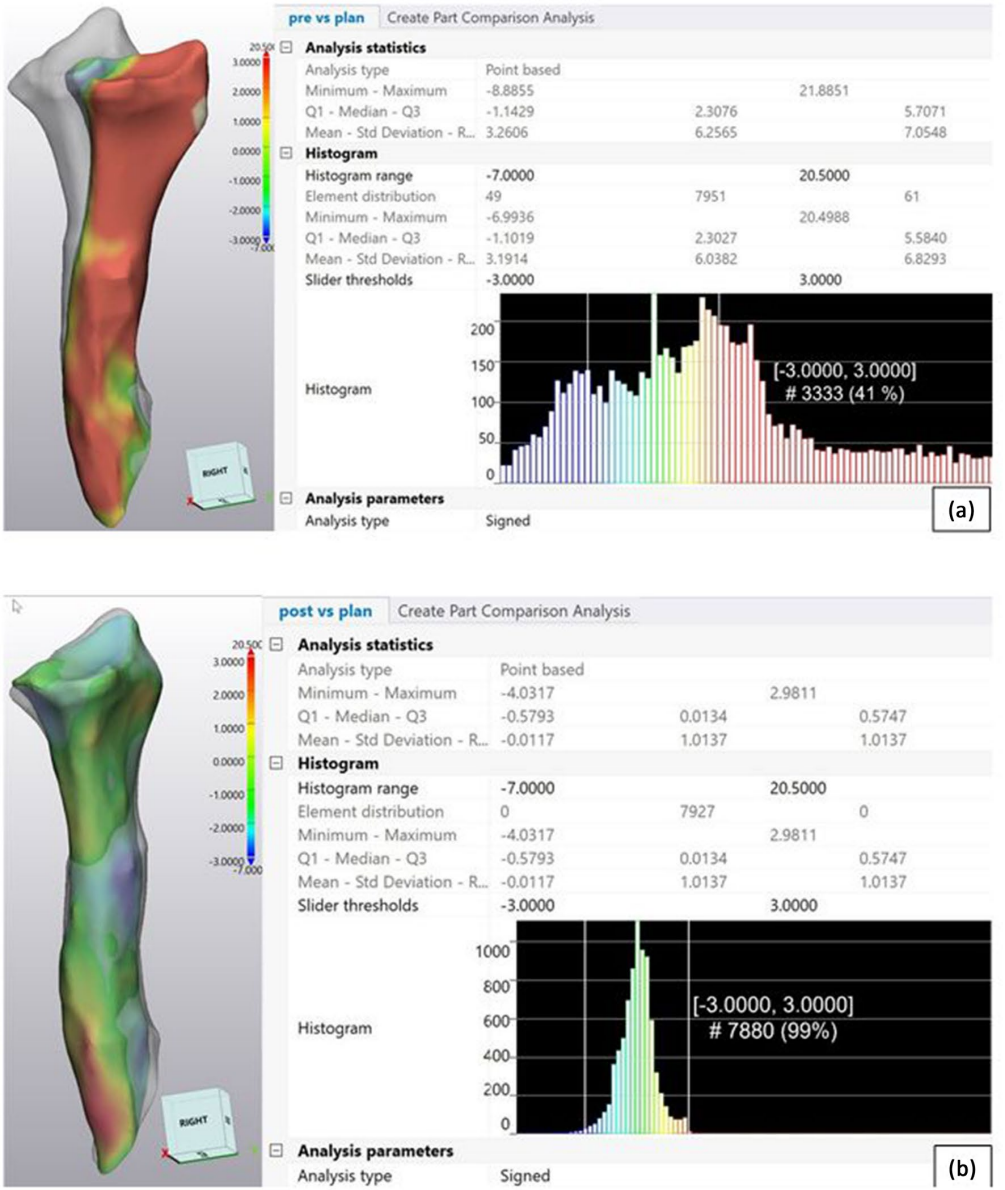
Part comparison analysis for the example of radius in case 5 (distances in mm). **a** Preoperative versus planned part: only 41% of surface points are situated in the range of − 3 mm to + 3 mm. The red colour of the preoperative part indicates a deviation of > 3 mm from the planned part. **b** Postoperative versus planned part. 99% of surface points are situated in the range of − 3 mm to + 3 mm. Green colour of the postoperative part shows the same position as the two parts. The analysis statistics and histogram show the minimum and maximum deviations of the two parts, the mean value of the point deviations, and the statistical dispersion of the same

We used the “Part Comparison Analysis” tool of the Mimics Innovation Suite software of Materialise to quantify 3D accuracy. This approach uses the iterative closest point (ICP) algorithm, which measures point deviations of the surface of bone parts. It provides point-based analysis statistics with the minimum and maximum deviation value in [mm] of both parts as mean, standard deviation (SD), and root mean square (RMS) of all deviations of surface points in [mm] of both parts (Fig. 4).

Furthermore, we determined a slider threshold in the histogram from − 3 mm to  + 3 mm. Thus, we obtained the number of values, in percent, that is included in this range [21]. Based on the generally accepted 2 mm tolerance for intraarticular dislocations in the context of fracture treatment, we defined the target range of ± 3 mm deviation in extraarticular areas as acceptable. In this way, we obtained one value in percent that indicates the match between the preoperative 3D planning and the postoperative 3D outcome, which we could correlate with the clinical result.

### Statistical analysis

IBM software SPSS Statistics 27 was used for descriptive statistics and statistical tests. The sign test has been applied to test consistent differences between VAS and DASH before and after treatment. The Wilcoxon test was carried out to test part comparison values between group “planned vs after surgery” and group “before surgery vs planned”. Spearman’s correlation was used to examine the relation between accuracy and ΔVAS, respectively, also ΔDASH.

## Results

### Patient-reported postoperative evaluation

Three patients were very satisfied, two patients were satisfied, three patients were neither satisfied nor dissatisfied, and no patient was dissatisfied or very dissatisfied with the result of surgery. The average VAS score significantly decreased from 6.5 ± 4.1 cm preoperatively to 2.3 ± 2.6 cm at the follow-up time point (*p* = 0.008, exact significance one sided). The average DASH score significantly improved, from 48.4 ± 30.9 to 27.0 ± 25.2 (*p* = 0.015, exact significance one sided) (detailed patient data in Appendix, Table 4).

### Clinical postoperative evaluation

At follow-up in side comparison of ROM of the elbow, forearm, and wrist in some cases were restricted on the affected side until 30°, with the forearm turning even in one patient until 90° (for detailed data see Appendix Tables 5 and 6).

The measured value of grip strength at the time of follow-up was reduced in 4 cases to a value in a range of 58% to 74% of the lowest value of the normal range (detailed data in Appendix Table 7).

### Part comparison analysis and clinical outcome

It was found that significantly more points in the comparison “planned vs after surgery” were in the range of the − 3 mm to 3 mm interval than before surgery: 87.3 ± 13.8% vs 48.9 ± 16.6%, respectively (Table 2). In 5 of 9 cases, this value was higher than 90%, of which 3 were 99%. In 2 cases, the values of 79% and 88% seemed still acceptable, and in one case with the correction of 2 bones; however, with 69% and 63%, it was out of the target range. Overall, the maximum deviation value was 4.5 ± 1.1 mm, and the minimum deviation value was − 4.5 ± 1.2 mm, after surgery. SD and RMS also decreased significantly (Table 2). (Individual values of part comparison analysis are presented in Appendix Table 8).

**Table 2:** Statistics for part comparison analysis

	Planned vs postoperative	Preoperative vs planned
Maximum deviation value (mm)	4.5 ± 1.1 (*p* = 0.002)*	12.9 ± 6.2
Minimum deviation value (mm)	− 4.5 ± 1.2 (*p* = 0.020)*	− 7.2 ± 2.1
SD (mm)	1.7 ± 0.6 (*p* = 0.002)*	4.5 ± 1.5
RMS (mm)	1.8 ± 0.7 (*p* = 0.002)*	5.5 ± 2.4
Number of values situated in the range − 3 mm to 3 mm (%)	87.3 ± 13.8 (*p* = 0.004)^a^	48.9 ± 16.6

^a^Significant difference to “preoperative vs planned”

Clinically, in all cases with higher accuracy (> 90%), an improvement of either DASH or VAS or both of > 60% to the preoperative values occurred. Patients with accuracy > 90% reported all levels of satisfaction with the surgery: very satisfied, satisfied, and neither (Table 3). In Spearman’s correlation there was a significant correlation of accuracy (%) and ΔVAS of *p* = 0.004 (rho = 0.928), but not of accuracy (%) and ΔDASH (*p* = 0.478).

**Table 3:** Patient-reported postoperative evaluation and 3D accuracy

Patient	VAS (cm) before surgery	VAS (cm) after surgery	DASH before surgery	DASH after surgery	Patient satisfaction	Values within the range
						± 3 mm (%) after surgery
Elbow						
1	10	5.5	88	25	Satisfied	94 humerus
						96 ulna
Forearm						
3	1.8	1.7	33	30	Neither	69 radius
						63 ulna
5	10	0.4	58	24	Very satisfied	99 radius
						99 ulna
Distal radius						
6	3.6	0.9	71	24	Satisfied	79
7	7.5	7.0	81	81	Neither	88
8	8.7	2.8	33	32	Neither	99

### Bone consolidation and complications

In all cases, there were no problems with prolonged bone healing. In all cases, bone consolidation was completed at the end of the follow-up period during our outpatient aftercare. There were no method-related complications. Patient 7 suffered from a peri-implant fracture in the area of the proximal plate end after a fall. So, the osteosynthesis was renewed with the same but longer plate. The plate holes distal to the osteotomy and the distal holes of the proximal fragment were used again so that the correction could, in principle, be retained. This probably resulted in a minor correction loss.

## Discussion

In the present case series, 12 osteotomies in 11 bones in 8 patients were re-examined after complex corrective upper extremity osteotomies, which were carried out with the help of computer-planned and 3D-printed PSIs.

No patient was dissatisfied with the result of the procedure, and the VAS and DASH scores improved significantly. In the part comparison analysis “planned vs postoperative comparison”, significantly more points in percent (= 3D accuracy) were in a − 3 mm to 3 mm interval than in the “preoperative vs planned comparison”. With the "part comparison analysis" tool, which is presented in this context here for the first time, it is quite possible to visualize the existing deviations and present them quantitatively in one value. In 3 cases a 3D accuracy of > 90% was achieved, in 2 cases it was in the range between 70 and 90%, and in one case it was < 70%. Clinically, in all cases with higher accuracy (> 90%), an improvement of either DASH or VAS or both of > 60% to the preoperative values occurred. Despite the small number of cases, there was a significant correlation between accuracy (%) and ΔVAS. Based on a limited number of cases, the study shows a possible way to calculate the correction accuracy using pre- and postoperative standardized CTs and correlate them with clinical parameters.

However, in some cases, ROM and grip strength after surgery did not fully achieve those of the opposite side despite intensive physiotherapy. From our point of view, this might be caused by the soft tissue situation and adaption, respectively. In the series, 4 patients were included whose injury had occurred many years previously, and 2 patients whose malalignment was congenital. With this surgical method, it is possible to correct the bony situation anatomically, but the soft tissues, joint capsules, and especially the muscles initially remain unchanged. Any persisting functional restrictions could be, at least partially, attributed to these factors. Accordingly, we preoperatively did not expect a function completely identical to the opposite side. Based on this, surgical goals were formulated with the patients, which were mainly fulfilled according to patients’ satisfaction statements. On the other hand, our results indicate that high 3D accuracy (> 90%) is also associated with obvious improvements in DASH and VAS. Vroemen et al. [5] also supported this correlation between clinical outcome and 3D accuracy in their study. In this context, they were able to show by postoperative 3D measurements that after corrective osteotomies of the distal radius, which were planned using 2D X-ray images, 3D rotational deficits were negatively correlated with the clinical outcome. Based on these and our results, CT-based procedures with postoperative 3D analysis seem to be necessary for the future to achieve optimal correction results.

An exciting question of this case series was how exactly the planned bone position was achieved by the surgical procedure. There was a significant reduction of deviation values in part comparison analysis after surgery and a significant increase in the number of points in the target interval (± 3 mm) in the histogram. However, not in all cases did we achieve an accuracy of > 90%. With some experience with the method, we attribute this to the more difficult exact positioning in diaphyseal cases, in which the bone is uniformly cylindrical and does not offer as many landmarks for PSI placement as on the distal humerus or radius. This might be associated with an increased risk of minimally incorrectly placed PSIs in this region. In case 5, also a diaphyseal case, a correction with 99% of the points in the ± 3 mm interval was achieved. This might be explained by the fact that the plate holes of the previous operation were available as a reference. Understandably, the method brings exact correction results only if the PSI positioning is carried out as planned. We also noticed the following possible sources of error. First, some plans contain pre-bent plates. Pre-bending is carried out using the outcome models provided. The slightest deviations in pre-bending also lead to correction deviations. Furthermore, it is not always possible to pull the plate exactly onto the bone with locking screws. With the use of reduction forceps or non-locking screws, which we, therefore, partly included in the planning, the plate can also be slightly bent again during reduction. Alternative planning with space between bone and plate and complete sets of locking screws does not completely solve the problem either, since the exact spaces have to be set precisely during the reduction. If all these small sources of error are taken into account, excellent corrections can be achieved with experience. When planning the guide, fixed reference points, e.g. existing plate holes or prominent bone protrusions, should be specified for the initial placement.

In postoperative 3D analyses of other groups, however, similar deviations to our results were reported, and similar reasons for this were filtered out. Omori et al. [6] examined the postoperative accuracy of 3D corrective osteotomy for cubitus varus deformities with custom-made surgical guides based on computer simulation for 17 patients. Error in the corrective surgery was calculated by the surface registration technique and 6 degrees of freedom based on the local coordinate system by the Euler angle method. They also used different software and another method for PSI manufacturing. The group presented mean errors of 7.1 ± 6.3 mm in proximal–distal translation. They discuss the loss of correction during internal fixation as a possible explanation. Vlachopoulos et al. [12] examined 3D postoperative accuracy in 14 patients after extraarticular forearm osteotomies using CT scan-based patient-specific surgical guides. The residual deformity was quantified in all 6 degrees of freedom. The residual rotation was expressed in axis–angle representation and additionally as 3 constitutive rotations (i.e. Euler rotations) around a standardized coordinate system. The residual translation was expressed as a 3D vector describing the displacement concerning the same coordinate axes. In opening wedge osteotomies, they observed a quite large residual rotational deformity of 8.30° ± 5.35°. They discussed reduction loss in cases with the high tension of soft tissue and more difficult guide fitting in shaft regions with a more circular shape, like us. Nevertheless, they concluded that all residual deformities were considerably smaller compared to corrective osteotomies performed without patient-specific guides. Stockmans et al. [10] reported their results in 4 patients after virtual planning and PSIs for a combined intra- and extraarticular malunion of the distal radius. For the extraarticular malunion, the 3D volar tilt, 3D radial inclination, and 3D ulnar variance were measured before and after surgery. For this purpose, reference points similar to the posterior–anterior and lateral X-ray were placed in the 3D model (e.g. the volar and dorsal lip of lunate fossa, most distal point of radial styloid, and most proximal point on the rim of the lunate fossa). The difference between planned and postoperative volar tilt was − 6° ± 6°. Also, in radial inclination, they had a greater difference and standard deviation of − 1° ± 5°. For the evaluation of intraarticular malunion, distance map measurements were used. In the histogram, maximal deviations of 3.4 mm in some intraarticular areas between planned and postoperative 3D surfaces were documented. Thus, this group concluded that there is a tendency to achieve higher accuracy as experience builds up, both on the surgeon’s side and on the design engineering side.

The limitation of the study is the small number of cases and its retrospective character. Determining the 3D accuracy of the two patients with very good satisfaction and the best clinical outcome (VAS and DASH 0) was not possible due to the lack of postoperative CT scans. The meaningfulness of statistical tests with values of 6 cases seems limited although significant in some aspects. One reason for a limited number of cases is the time and financial expenditure of the method with costs per case of about 2500 up to 3800 Euro and time of about 2 additional hours for the surgeon who plans it with help of an engineer.

## Conclusion

Based on the findings with good patient satisfaction, clinical outcome, and 3D-radiological results, we will continue to use the method for more complex adjustment osteotomies on the upper extremity despite its high financial and time expenditure especially when there is no alternative surgical approach. Expanding experience with and further development of the procedure will potentially lead to even more consistent matching between planned and postoperative 3D models and thus to the full exploitation of the maximum surgical possibilities in these cases. Examination of the 3D accuracy is, therefore, required for the further development of the method. Since the data indicate a positive correlation between 3D accuracy and clinical outcome, postoperative 3D analysis is required to be able to carry out the operative revision promptly if necessary. From our point of view, the “Part comparison tool” is an easy-to-use software tool for quantifying 3D accuracy. Further studies are needed to define norm ranges for each bone that correlate with the clinical outcome and that indicate when a surgical revision is recommended. In conclusion, the method has the potential to become a standard procedure in reconstructive orthopaedic surgery in the case of upper extremity deformities.

## Data Availability

All essential test results are given in the tables in Appendix.

## Appendix

See Tables 4, 5, 6, 7, and 8.

**Table 4:** Patient-reported postoperative evaluation

Patient	VAS (cm)	VAS (cm)	DASH	DASH	Patient
	Before surgery	After surgery	Before surgery	After surgery	Satisfaction
Elbow					
1	10	5.5	88	25	Satisfied
2	10	0	23	0	Very satisfied
Forearm					
3	1.8	1.7	33	30	Neither
4	0	0	0	0	Very satisfied
5	10	0.4	58	24	Very satisfied
Distal radius					
6	3.6	0.9	71	24	Satisfied
7	7.5	7.0	81	81	Neither
8	8.7	2.8	33	32	Neither

**Table 5:** ROM of elbow and forearm in side-by-side comparison at time of follow-up (side-different values in bold)

Patient	Side of surgery	Extension/flexion elbow [°]			Supination/pronation forearm [°]		
		Right	Left	Difference	Right	Left	Difference
Elbow							
1	Left	0/0/135	0/10/135	**0/− 10/0**	90/0/90	85/0/85	**− 5/0/− 5**
2	Left	0/0/135	0/0/135	0/0/0	90/0/90	90/0/90	0/0/0
Forearm							
3	Right	0/10/140	10/0/130	**− 10/− 10/10**	0/0/90	90/0/90	**− 90/0/0**
4	Left	10/10/130	10/10/140	**0/0/10**	70/0/90	80/0/90	**10/0/0**
5	Right	5/0/130	5/0/130	0/0/0	70/0/65	80/0/80	**− 10/0/− 15**
Distal radius							
6	Left	0/0/130	0/0/130	0/0/0	90/0/90	70/0/80	**− 20/0/− 10**
7	Left	0/0/145	0/0/145	0/0/0	90/0/80	90/0/80	0/0/0
8	Right	10/0/140	10/0/140	0/0/0	90/0/90	90/0/90	0/0/0

**Table 6:** ROM of wrist in side-by-side comparison at time of follow-up (side-different values in bold)

Patient	Side of surgery	Wrist extension/flexion [°]			Wrist radial/ulnar abduction [°]		
		Right	Left	Difference	Right	Left	Difference
Elbow							
1	Left	40/0/40	50/0/60	**10/0/20**	20/0/30	20/0/30	0/0/0
2	Left	60/0/70	60/0/70	0/0/0	30/0/40	30/0/40	0/0/0
Forearm							
3	Right	40/0/40	70/0/60	**− 30/0/− 20**	10/0/35	20/0/40	**− 10/0/− 5**
4	Left	60/0/70	60/0/70	0/0/0	15/0/40	15/0/35	**0/0/-5**
5	Right	30/0/40	40/0/60	**− 10/0/− 20**	20/0/30	30/0/40	**− 10/0/− 10**
Distal radius							
6	Left	60/0/50	30/0/20	**− 30/0/− 30**	30/0/40	20/0/30	**− 10/0/− 10**
7	Left	55/0/55	50/0/40	**− 5/0/− 15**	10/0/30	30/0/30	**20/0/0**
8	Right	30/0/20	40/0/30	**− 10/0/− 10**	20/0/30	20/0/30	0/0/0

**Table 7:** Grip strength (in pounds). Range age-, sex-, and handedness-dependent clinical norms in brackets [15]

Patient	Side of surgery	Right	Left
Elbow			
1	Left	83 (76–176)	**54 (73–157), 74%**
2	Left	88 (64–172)	77 (54–149)
Forearm			
3	Right	**43 (64–172), 67%**	60 (54–149)
4	Left	78 (49–108)	85 (41–94)
5	Right	**41 (65–155), 63%**	90 (58–160)
Distal radius			
6	Left	67 (39–100)	49 (37–83)
7	Left	37 (37–77)	**17 (29–66), 58%**
8	Right	60 (30–93)	50 (26–73)

Right-handedness of all patients. Deviations from the norm in bold. In these cases, the measured value from the lowest value of the clinical norm is provided (percent)

**Table 8:** Individual values of part comparison analysis

		Maximum deviation value (mm)	Minimum deviation value (mm)	Standard deviation (mm)	RMS (mm)	Values in the range **−** 3 mm to 3 mm (%)
Elbow						
Case 1	Humerus plan vs post	3.8	**−** 5.3	1.6	1.6	94
	Humerus pre vs plan	16.7	**−** 11.7	5.0	5.2	58
	Ulna plan vs post	5.2	**−** 2.8	1.1	1.2	96
	Ulna pre vs plan	9.0	**−** 7.4	3.4	3.9	50
Forearm						
Case 3	Radius plan vs post	6.1	**−** 5.7	2.6	2.8	69
	Radius pre vs plan	13.5	**−** 6.0	4.9	6.2	46
	Ulna plan vs post	5.8	**−** 5.3	2.6	2.7	63
	Ulna pre vs plan	21.7	**−** 4.9	6.7	10.2	31
Case 5	Radius plan vs post	3.0	**−** 4.0	1.0	1.0	99
	Radius pre vs plan	21.9	**−** 8.9	6.3	7.1	41
	Ulna plan vs post	3.3	**−** 3.9	1.1	1.1	99
	Ulna pre vs plan	12.7	**−** 5.4	4.5	6.4	39
Distal Radius						
Case 6	Radius plan vs post	5.0	**−** 5.0	2.2	2.2	79
	Radius pre vs plan	8.9	**−** 6.3	4.6	5.1	29
Case 7	Radius plan vs post	5.1	**−** 5.7	1.8	1.9	88
	Radius pre vs plan	5.1	**−** 7.8	2.4	2.4	80
Case 8	Radius plan vs post	3.6	**−** 2.8	1.3	1.3	99
	Radius pre vs plan	6.2	**−** 6.9	2.8	2.9	66

## Abbreviations

2D: Two-dimensional
3D: Three-dimensional
CT: Computed tomography
DASH: Disabilities of arm, shoulder, and hand
ICP: Iterative closest point
PSI: Patient-specific instrument
RMS: Root mean squared
ROM: Range of motion
SD: Standard deviation
VAS: Visual analogue scale
